# Tanshinones suppress AURKA through up-regulation of miR-32 expression in non-small cell lung cancer

**DOI:** 10.18632/oncotarget.3933

**Published:** 2015-05-14

**Authors:** Zhong-Liang Ma, Bing-Jie Zhang, De-Tao Wang, Xue Li, Jia-Li Wei, Bo-Tao Zhao, Yan Jin, Yan-Li Li, You-Xin Jin

**Affiliations:** ^1^ School of Life Sciences, Shanghai University, Shanghai 200444, China; ^2^ Institute of Biomedicine and Biotechnology, Shenzhen Institutes of Advanced Technology, Chinese Academy of Sciences, Shenzhen 518055, China; ^3^ State Key Laboratory of Molecular Biology, Institute of Biochemistry and Cell Biology, Institutes for Biological Sciences, Chinese Academy of Sciences, Shanghai 200031, China

**Keywords:** tanshinone, AURKA, miRNA, non-small cell lung cancer

## Abstract

Tanshinone is the liposoluble constituent of *Salia miltiorrhiza*, a root used in traditional herbal medicine which is known to possess certain health benefits. Although it is known that tanshinones, including tanshinone I (T1), tanshinone IIA (T2A), and cryptotanshinone (CT), can inhibit the growth of lung cancer cells *in vitro*, the mechanism under which they act is still unclear. AURKA, an oncogene, encodes a serine-threonine kinase which regulates mitotic processes in mammalian cells. Here, we reported that tanshinones mediate AURKA suppression partly through up-regulating the expression of miR-32. We found that tanshinones could inhibit cell proliferation, promote apoptosis, and impede cell-cycle progression, thus performing an antineoplastic function in non-small cell lung cancer (NSCLC). Additionally, we demonstrated that tanshinones attained these effects in part by down-regulating AURKA, corroborating previous reports. Our results showed that in NSCLC, similar effects were obtained with knock-down of the AURKA gene by siRNA. We also verified that AURKA was the direct target of miR-32. Collectively, our results demonstrated that tanshinones could inhibit NSCLC by suppressing AURKA via up-regulating the expressions of miR-32 and other related miRNAs.

## INTRODUCTION

In recent years, there have been several advances in the treatment of lung cancer, notably a diversification in therapeutic drugs, as well as mode of treatment. Among these, targeted therapies have taken the forefront based on such features as efficiency and a low rate of adverse reactions [1–4]. Tanshinones, including tanshinone I (T1), tanshinone IIA (T2A), and cryptotanshinone (CT), are the liposoluble constituents of *Salia miltiorrhiza*, a traditional herbal therapy which has favorable medicinal value [5]. Of particular interest is tanshinone's cytotoxic effect on tumor cells, which is thought to act primarily through inducting apoptosis and interdicting cell cycle progression, angiogenesis, cell invasion and metastasis [6–9]. Related studies have indicated that tanshinones might effectively target human lung cancer cells and could inhibit growth by induction of apoptosis [6, 10–12]. Thus, it is reasonable to speculate that tanshinones would play an important role in the treatment of non-small cell lung cancer (NSCLC). Research shows that tanshinone molecules inhibit the growth of lung cancer cells *in vitro* [13] and that the mechanism may be via interruption of cell cycle progression and induction of cell apoptosis, resulting in down-regulation in the expression of cell-cycle related proteins, Aurora A and Cyclin B, as well as the apoptosis related protein Bcl2.

Aurora A is a serine-threonine kinase encoded by the AURKA (aurora kinase A) gene, which is responsible for regulating mitotic processes in mammalian cells, including centrosome maturation, spindle assembly, and chromosome segregation. Various types of cancers exhibit amplification of AURKA and serious effects such as chromosomal instability, centrosomal amplification/aneuploidy, therapeutic resistance, cell-cycle progression and anti-apoptosis are induced by overexpression of AURKA. Synergistically, these events promote the progression of cancer. Hence, AURKA can be considered an oncogene and thus an important target for cancer therapy.

MicroRNAs (miRNAs) are a class of single stranded small non-coding RNA. They are about 18–23 nucleotides (nt) in length, encoded by an endogenous gene, and regulate gene expression at the post-transcriptional level. Importantly, altered expression of miRNAs is reported in a variety of human cancers and may be associated with cancer pathogenesis, tumor growth, and metastasis [14, 15]. Because miRNAs play a regulatory role in the tumorigenesis process and can regulate the expression of tumor associated genes [15–17], we proposed that tanshinones may regulate the expression of AURKA via adjusting the expression of related miRNAs.

## RESULTS

### Tanshinones inhibit cell proliferation, promote apoptosis and impede cell-cycle progression in NSCLC

**Figure 1:**
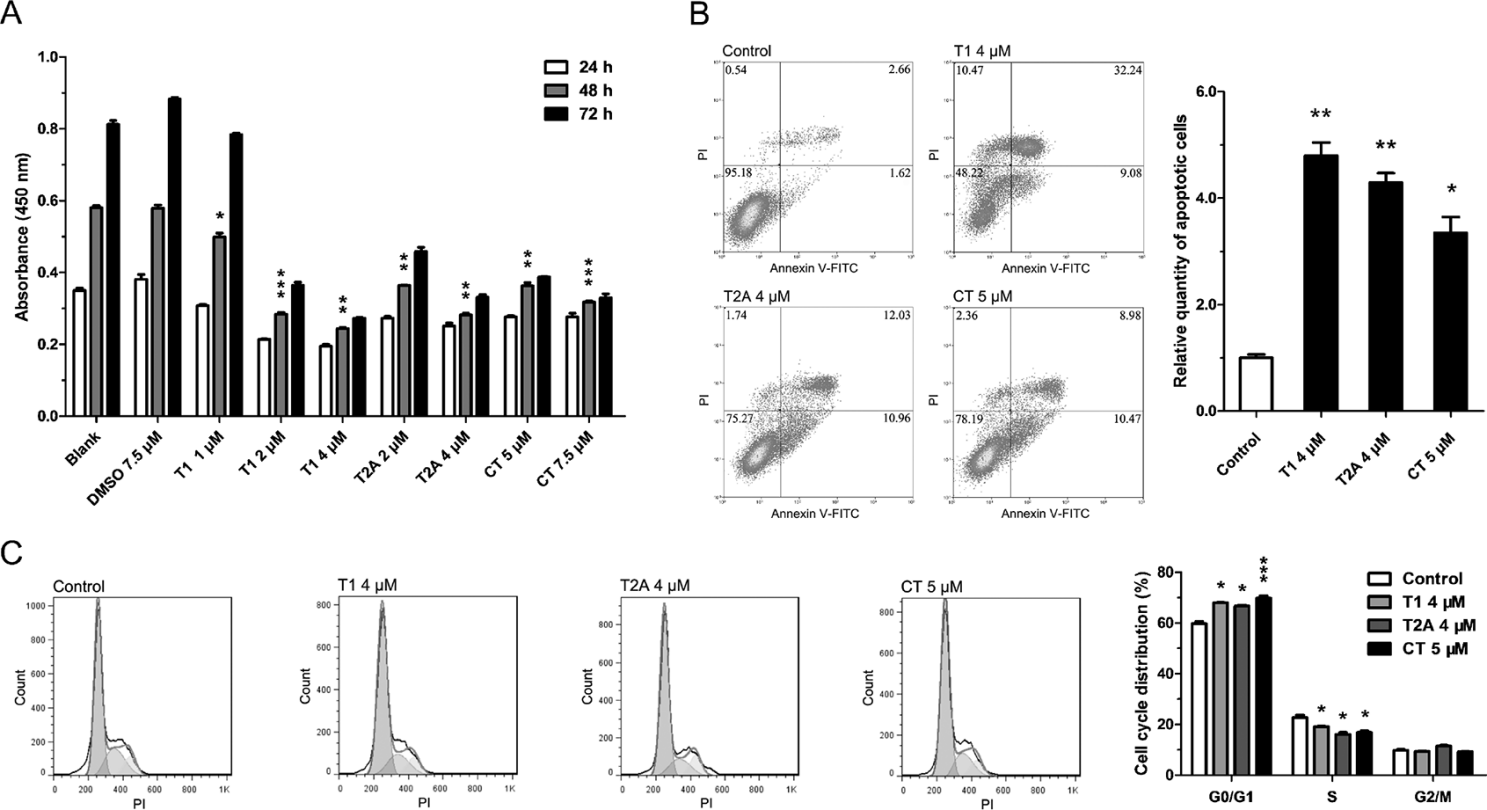
Tanshinone can suppress NSCLC **A.** Cell vitality of H1299 cells treated with tanshinone or DMSO was determined by CCK-8 cytotoxicity test. Results are represented as the mean±SEM of OD_450nm_. Blank serves as control. In **B–C.** H1299 cells were respectively treated with T1 4 μM, T2A 4 μM, CT 5 μM or DMSO 5 μM (Control) for 48 h. **B.** Cell apoptosis situations of H1299 cells were detected by flow cytometry. **C.** Cell cycle distributions of H1299 cells were detected by flow cytometry. *P < 0.05, **P < 0.01, ***P < 0.001, vs. control (*n* = 3). Representative of triplicate experiments was shown.

To verify the suppression role of tanshinones in NSCLC, we first measured its anti-proliferative effects in several NSCLC cell lines, such as H1299, A549, and SPCA-1. The results showed that tanshinones could inhibit the proliferation of NSCLC cells in a time- and dose-dependent manner (Figure 1A, Supplementary Figure S1A–S1B) and that cell proliferation was significantly inhibited by tanshinones at concentrations of 2 μM/4 μM for T1, 2 μM/4 μM for T2A, and 5 μM/7.5 μM for CT (*P* < 0.005/0.001) in H1299 cells (Figure 1A). In addition, results also indicated that T1 was the most effective of the tanshinones tested and that DMSO, the solvent of tanshinones, had no effect on cell proliferation.

The H1299 cell line was chosen to test the effects of tanshinones on apoptosis, cell cycle, and cell migration. The percentages of apoptotic cells in the tanshinone-treated groups were much higher than in the control (Figure 1B). Following treatment with tanshinones, the proportion of cells at the G0/G1 phase increased more than 10% as compared with the control (Figure 1C). Almost no difference was observed in the ability of cell migration between the experimental groups and the control group (Supplementary Figure S2). These results suggested that tanshinones could significantly promote apoptosis (Figure 1B) and cause G0/G1 cell-cycle arrest (Figure 1C) in H1299 cells. Thus tanshinones could exhibit an important antineoplastic effect in NSCLC tumor cells via inhibition of cell proliferation, promotion of apoptosis, and retardation of cell-cycle progression.

### Tanshinones inhibit NSCLC by down-regulating the expression of AURKA

**Figure 2:**
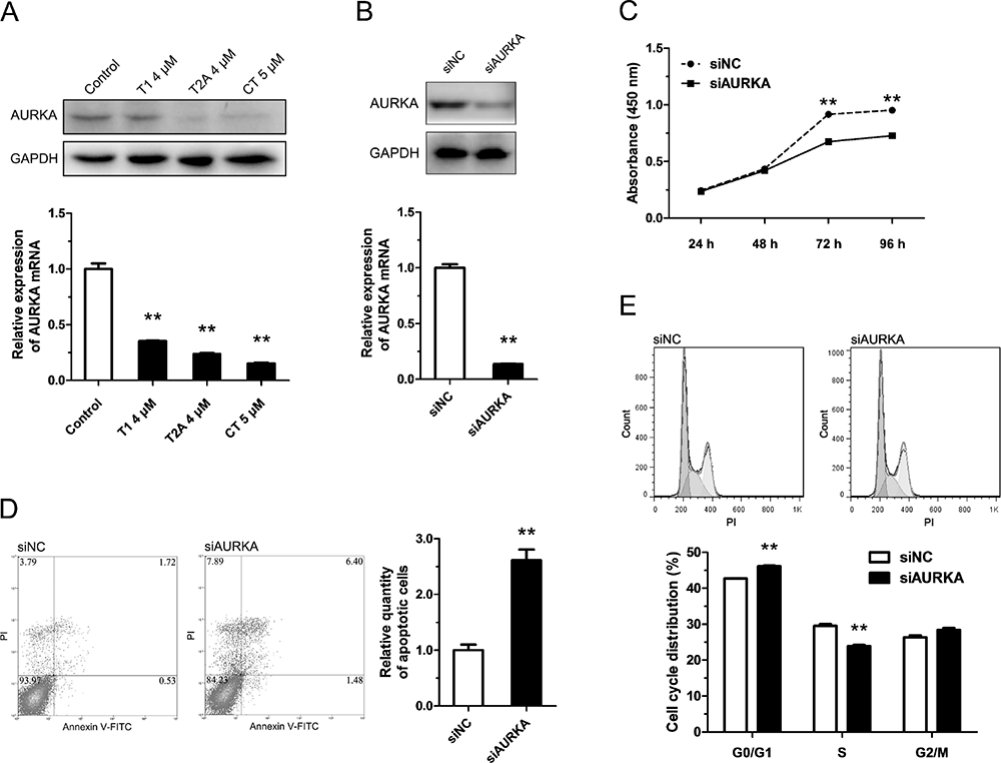
Tanshinone inhibits NSCLC by down-regulating the expression of AURKA **A.** The variation of mRNA and protein of AURKA in H1299 cells respectively treated with T1 4 μM, T2A 4 μM, CT 5 μM or DMSO 5 μM (Control) for 48 h were measured by qRT-PCR and Western blot. In **B–E.** H1299 cells were transfected with siAURKA or siNC (100nm) and incubated at 37°C for 24 h (96 h for CCK-8 cytotoxicity test ). **B.** The variation of mRNA and protein of AURKA in H1299 cells were measured by qRT-PCR and Western blot. **C.** Cell proliferation of H1299 cells were determined by CCK-8 cytotoxicity test. **D.** Cell apoptosis situations of H1299 cells were detected by flow cytometry. **E.** Cell cycle distributions of H1299 cells were detected by flow cytometry. **P < 0.01, vs. control (*n* = 3). Representative of triplicate experiments was shown.

Li et al. [13] found that, in NSCLC, the suppressive effect of tanshinones may be due partly to down-regulation of AURKA. To confirm this, we checked the variation of AURKA mRNA and protein after exposure to tanshinones (Figure 2A). Our data revealed that, in H1299 cells incubated for 48 h with 4 μM T1, 4 μM T2A, or 5 μM CT, the contents of AURKA mRNA and protein were much lower than the control group (DMSO 5 μM for 48 h) (Figure 2A). This result indicated that tanshinones could suppress the expression of AURKA. To further study the role of AURKA in NSCLC, we knocked down AURKA using siRNA and then surveyed the change in cell proliferation, apoptosis, and cell-cycle progression. After 24 h post-transfection with siAURKA/siNC, the content of AURKA mRNA decreased by almost 90% as compared to the control (Figure 2B). The Aurora A protein decreased about 60% after transfection for 24 h (Figure 2B). Results additionally showed that knocked-down of AURKA could suppress cell proliferation, accelerate apoptosis, and impede cell-cycle progression in an effect similar to that of tanshinones treatment (Figure 2C–2E). Besides, we measured the endogenous expression level of AURKA in common NSCLC cell lines and found that the expression level of AURKA in H1299 cells was almost 7 times of in BEAS-2B, control cells (Supplementary Figure S3). In SPCA-1, the expression level of AURKA was about treble of in BEAS-2B. There approximately no difference between in A549 and in BEAS-2B. This result could explain why H1299 cells were much more sensitive to tanshinones than SPCA-1 and A549 cells (Figure 1A, Supplementary Figure S1A–S1B). Based on the above-mentioned results, it is reasonable to surmise that tanshinones restrain NSCLC in part by down-regulating the expression of AURKA.

### Tanshinones suppress AURKA partly via up-regulating the expression of miR-32

**Figure 3:**
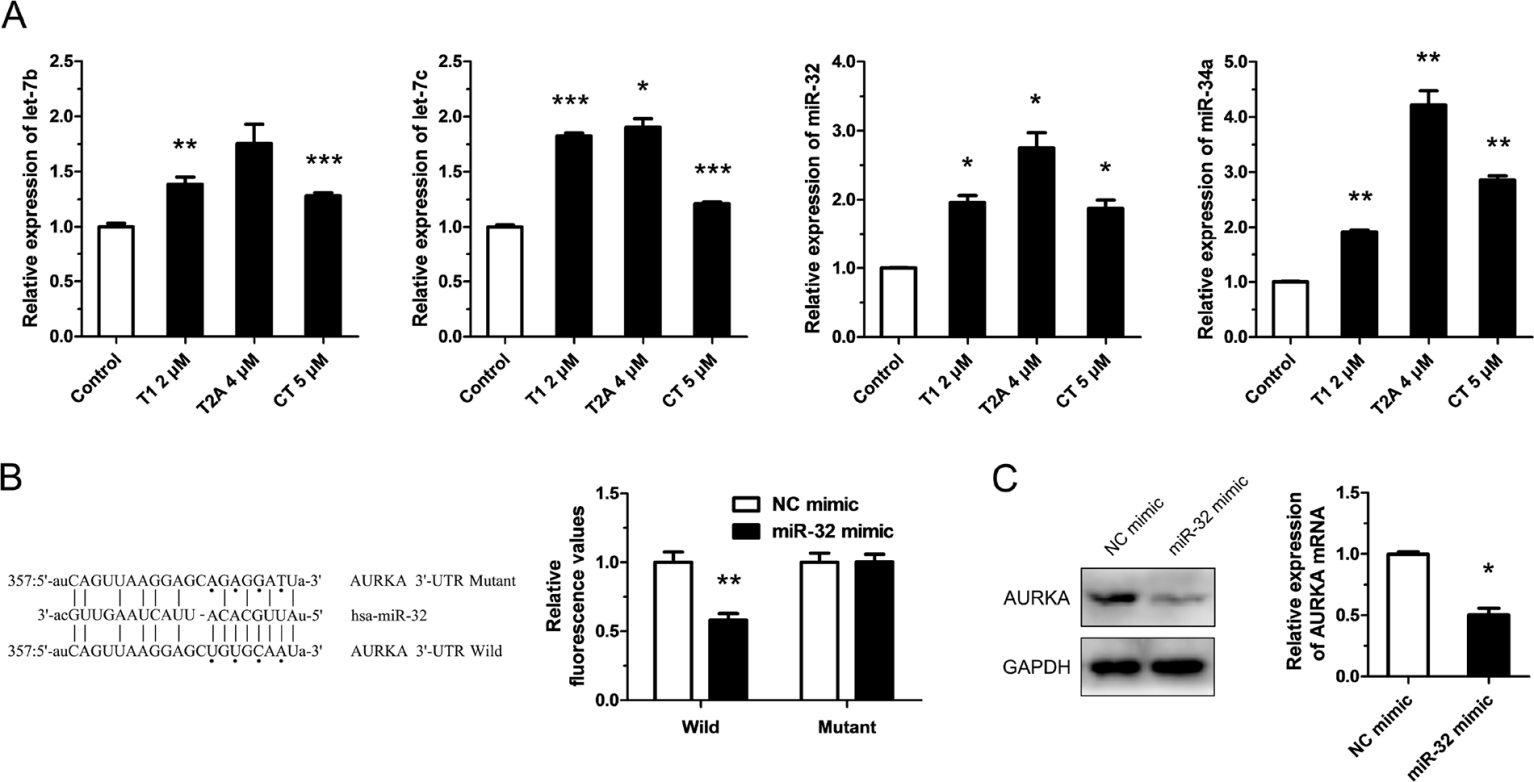
Tanshinone suppresses AURKA partly through up-regulating the expression of miR-32 **A.** Expression levels of miRNAs in H1299 cells respectively treated with T1 4 μM, T2A 4 μM, CT 5 μM or DMSO 5μM (Control) for 48 h were measured by qRT-PCR. **B.** Dual luciferase reporter assay. Luciferase reporter constructs containing wild-type or mutated AURKA 3′-UTR were cotransfected with miR-32 mimics or NC mimics into H1299 cells. Relative firefly luciferase expression was normalized to Renilla luciferase. **C.** The variation of mRNA and protein of AURKA in H1299 cells transfected with miR-32 mimics or NC mimics (100 nm) and incubated at 37°C for 24 h were measured by qRT-PCR and Western blot. *P < 0.05, **P < 0.01, ***P < 0.001, vs. control (*n* = 3). Representative of triplicate experiments was shown.

Although we know of the relationship between tanshinones and AURKA, the mechanism by which tanshinones regulate AURKA is still unclear. It has been well established that miRNAs regulate gene expression at the post-transcriptional level and that abnormal expression and regulation of miRNAs are critically involved in tumorigenesis and tumor progression. MiRNAs can regulate the expression of tumor associated genes and play a central role in the tumorigenesis process. Therefore, we propose that tanshinones may regulate the expression of AURKA by adjusting the expression of related miRNA. We searched for miRNAs that might target AURKA and select the most promising ones for further investigation by TargetScanHuman 6.2, StarBase v2.0, miRanda and http://microRNA.org (Supplementary Table 1). Although miR-34a was not part of the original list, we still tested it because of its explicit antitumor effect in lung cancer and its place as one of the miRNAs our group has researched [18]. Results from qRT-PCR assays showed that the expression levels of the miRNAs being tested (let-7b/c, miR-25, miR-32, miR-34a, miR-92a/b, miR-137, miR-363, and miR-367) were significantly up-regulated by tanshinones (T1, T2A, and CT) (Figure 3A, Supplementary Figure S4). We next focused on miR-32, as it has been reported that it may be an anti-oncogene in lung cancer but that its mechanism in NSCLC has yet to be brought to light [19–21]. We set out to unmask the target relationship between miR-32 and AURKA. Data from the luciferase reporter assay revealed that there was a miR-32 binding site at 3′-UTR (357-379 bp) of AURKA (Figure 3B). Our results further showed that miR-32 mimic could down-regulate the expressions of AURKA mRNA and protein (Figure 3C). These data suggested that miR-32 targeted AURKA and that tanshinones suppressed AURKA by regulating the expression levels of miR-32 and other interrelated miRNAs.

### miR-32 acts as a tumor suppressor gene in NSCLC

**Figure 4:**
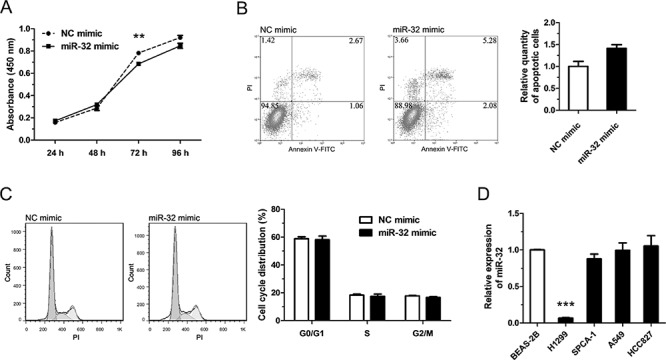
miR-32 plays a rule of tumor suppressor gene in NSCLC In **A–C**, H1299 cells were transfected with NC mimics or miR-32 mimics (100 nm) and incubated at 37°C for 48 h (96 h for CCK-8 cytotoxicity test). **A.** Cell proliferation of H1299 cells were determined by CCK-8 cytotoxicity test. **B.** Cell apoptosis situations of H1299 cells were detected by flow cytometry. **C.** Cell cycle distributions of H1299 cells were detected by flow cytometry. **D.** The expression levels of miR-32 in lung cancer cell lines or pulmonary epithelial cell line (control) were measured by qRT-PCR. **P < 0.01, ***P < 0.001, vs. control (*n* = 3). Representative of triplicate experiments was shown.

Previous studies [19–21] indicated that miR-32 may be a tumor suppressor gene and our results confirmed that it could inhibit cell proliferation, promote apoptosis of tumor cells in NSCLC, but that it had no obvious effect on cell-cycle progression (Figure 4A–4C). We found that the antitumor effect of miR-32 was not conspicuous in H1299 cells, but we also measured the endogenous expression level of miR-32 in the other common NSCLC cell lines and found that the expression level of miR-32 in H1299 cells was much lower compared to BEAS-2B, control cells (Figure 4D). Jalava et al reported that androgen receptor (AR) could regulate miR-32 expression by binding ARBS (AR-binding site) near the miR-32 genomic location [22]. Our results demonstrated tanshinones treatment can down-regulate the expression of AR, and at the same time the expression of miR-32 is up-regulated in NSCLC (Supplementary Figure S5). When considering that one miRNA may target several genes and that a single gene could be the target of multiple miRNAs [23–27] along with negative feedback mechanisms that can exist in cells [28–30], it's reasonable to assume that one single miRNA may not have a remarkable effect on tumor suppression or promotion. According to these results as well as previous research, we concluded that miR-32 could play the role of tumor suppressor in NSCLC.

## DISCUSSION

The value of tanshinones is well-known in both traditional Chinese medicine and modern medicine. As stated before, tanshinones have a cytotoxic effect on tumor cells via inducing apoptosis, arresting cell-cycle progression, restraining angiogenesis, suppressing invasion and metastasis. In the present study, we proved that tanshinones (T1, T2A and CT) could significantly inhibit the proliferation of NSCLC cell lines *in vitro* via inducing apoptosis and retarding cell-cycle progression. These attributes might make it an ideal medicine for NSCLC treatment. Because there were still many details yet unknown about the primary mechanism of the suppression of NSCLC by tanshinones, it was necessary to validate whether tanshinones exhibited toxicity and if there was the presence of any by-effects on normal human cells.

AURKA is a member of a novel oncogenic family of mitotic serine/threonine kinases. Abundant evidence suggests a role for AURKA in centrosome maturation [31], spindle formation [32], and G2-M transition [33]. AURKA is frequently over expressed in different types of cancer [34–37] and suppression of AURKA expression and function reduces tumor growth [38–40]. Tumorigenesis is strongly related to abnormal amplification and expression of AURKA, which has led to the recognition of AURKA as an important molecular target for cancer therapy [41, 42]. Our studies showed that tanshinones down regulated the expression level of AURKA *in vitro* (Figure 2A, Supplementary Figure S6). These results suggest that AURKA is a novel and important molecular target for tanshinones.

The interrelation between tanshinones and AURKA has been preliminarily investigated, but the mechanism by which tanshinones up-regulate the expression of AURKA is still unclear. Herein, we validated the regulatory relationship between tanshinones and AURKA and defined the effects of AURKA in NSCLC. Above all, we found that tanshinones suppressed AURKA through regulating the expression levels of miR-32 and other interrelated miRNAs, and that AURKA is the direct target of miR-32. Nevertheless, the mechanism for regulation of miRNAs by tanshinones is almost utterly ignorant. In the near future, we hope to commence investigation on this topic.

In summary, our study identified a novel network delineating the method by which tanshinones suppress NSCLC, first linking tanshinones with miRNAs and preliminary expounding the mechanism for this in NSCLC. Scientifically, this finding suggests tanshinones could efficiently be used as a targeted therapy in the treatment of NSCLC. Clinically, this study suggests AURKA as an ideal target for inhibiting cancer progression in NSCLC patients.

**Figure 5:**
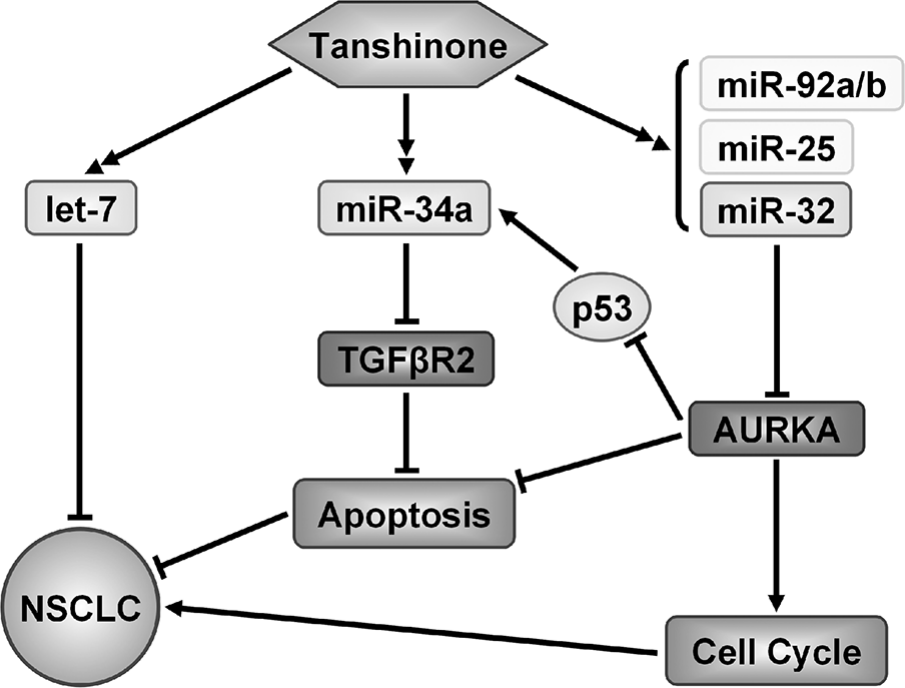
Model of identified mechanism how tanshinone suppresses NSCLC Tanshinone played a role of tumor suppressor in NSCLC by up-regulating several miRNAs to suppress genes which related to the cancer process.

Based on our results thus far, we were able to summarize the mechanism by which tanshinones suppress NSCLC (Figure 5). Tanshinones can up-regulate the expression levels of tested miRNAs (Figure 3A, Supplementary Figure S4, Supplementary Figure S6), among these is let-7, a well-known tumor suppressor that can inhibit the development of lung cancer [43–45]. Our previous research revealed that miR-34a targets TGFβR2 which inhibits apoptosis in NSCLC [46] and in recent years, many studies have verified that miR-34a could inhibit tumor progression in lung cancer [47, 48]. It was reported that miR-25, miR-32 and miR-92a/b are in the same miRNA family [49] and based on this, they may play roles in a specific cancer. miR-25 inhibition led to autophagic cell death by directly increasing ULK1 expression in breast cancer cell [50]. MiR-32 inhibits osteosarcoma cell proliferation and invasion by targeting Sox9 [20], however miR-92a/b as oncogene, promotes cell proliferation and invasion in cancer [24, 51]. miR-25 family plays muti-roles in cancergenisis, we first report that T1 promotes miR-25 family and suppress the growth of NSCLC.

Our results demonstrated that miR-32 can suppress NSCLC by targeting AURKA (Figure 3B/3C, Figure 4) and it has been published that AURKA can suppress the effects of p53 [52, 53], an anti-oncogene which can up-regulate the expression level of miR-34a [54]. We also checked the the variation of AURKA/p53 mRNA and related miRNAs after exposure to tanshinones (Supplementary Figure S6). The result showed that, in SPCA-1 cells incubated for 48 h with 4 μM T1, 6 μM T2A, the content of AURKA mRNA was much lower than the control group (DMSO 6 μM for 48 h) (Supplementary Figure S6). To the contrary, the contents of p53 mRNA and miRNAs were mucher higher than control group. This result indirectly proved that tanshinones could abolish the AURKA mediated p53 suppression and up-regulate the expressions of related miRNA. Comprehensively, tanshinones suppress NSCLC mainly through up-regulating anti-oncogene miRNAs to suppress their target genes that promote cancer progression, therefore giving tanshinones the ultimate effect of promoting apoptosis and arresting cell-cycle (Figure 5).

## MATERIALS AND METHODS

### Chemicals and reagents

Tanshinones (T1, T2A, and CT) were obtained from Nanjing University (Nanjing, China) and dissolved in dimethyl sulfoxide (DMSO). The U6 and relevant miRNAs primers (Supplementary Table 1) were designed and made by Invitrogen (Shanghai, China). The CellTiter 96^®^ AQueous One Solution Cell Proliferation Assay was purchased from Promega (Beijing, China) and the Annexin V-FITC Apoptosis Detection Kit was purchased from BD Biosciences (USA). The One Step PrimeScript^®^ miRNA cDNA Synthesis Kit and SYBR Premix Ex Taq II (both, Perfect Real Time) were purchased from Takara (Dalian, China). All chemical reagents were purchased from Sangon Biotech (Shanghai, China) or Sinopharm Chemical Reagent (Shanghai, China).

### Cell culture

The human NSCLC cell line H1299 was obtained from ATCC. The human NSCLC cell lines A549, SPCA-1, HCC827 and BEAS-2B cell line which isolated from normal human bronchial epithelium were obtained from the Cell Bank, China Academy of Sciences (Shanghai, China). The HEK-293T cell line was obtained from the Cell Bank as well. BEAS-2B cells were cultured in LHC-9 medium (Gibco, Gaithersburg, USA). NSCLC cell lines were cultured in either RPMI 1640 or DMEM medium (Gibco). All the media were supplemented with 10% (v/v) fetal bovine serum (FBS). Cells were cultured at 37°C in a 5% CO_2_ atmosphere.

### Transfection

H1299 cells were transiently transfected with 100 nM of the chemically synthesized miR-32 mimic, negative control mimic (NC), miR-32 inhibitor, negative control inhibitor (NCi), siAURKA (sequence: 5′-AUGCCCUGUCUUACUGUCATT-3′), or negative control siRNA (siNC) (Ribobio, Guangzhou, China) using Lipofectamine 2000 (Invitrogen) according to the manufacturer's recommendations. After 24 to 48 h post-transfection, cells were used for subsequent experiments including assays for proliferation, western blot, apoptosis and cell cycle analysis.

### Cell proliferation analysis

Cell proliferation was assessed by Cell Counting Kit-8 (CCK-8) assay kit (Dojindo, Japan). Cells treated with tanshinones (T1 at 1, 2, and 4 μM; T2A at 2, 4, and 6 μM; CT at 5 and 7.5 μM), mimic, inhibitor, or siRNA were plated in a 96-well microplate (Corning Incorporated, Shanghai, China) and incubated at 37°C in a 5% CO_2_ atmosphere. Data were obtained from the measurement of 4 replicate wells for each data point. After incubation for 24, 48, 72 or 96 h, 10 μl of CCK-8 solution was added to the appropriate wells and incubated for 60 min. The absorbance was then measured by a multi-function enzyme-linked analyzer, FLx8 (BioTek, Shanghai, China), at the wavelength of 450 nm (OD, cell survival state).

### RNA isolation, reverse transcription and quantitative real-time PCR (qRT-PCR)

Following the manufacturers' instructions, total RNA was isolated using Trizol Reagent (Sangon Biotech, Shanghai, China) and cDNA synthesis was performed with the PrimeScript™ 1st Strand cDNA Synthesis Kit or SYBR^®^ PrimeScript™miRNA RT-PCR Kit (TaKaRa). Quantitative RT-PCR analysis was performed using SYBR Green II (TaKaRa a) on a CFX96™ Real-time System (Bio-Rad, Shanghai, China) according to the manufacturer's protocol. The expression levels of miRNAs were normalized to the U6 expression level. The expression levels of mRNAs were normalized to the 18S expression level.

### Cell apoptosis analysis

Cell apoptosis was determined by the Annexin V-FITC/propidium iodide (PI) apoptosis detection kit (BD Biosciences) following the manufacturer's protocol. Cells (1 × 10^5^ cells) were collected by trypsinization and centrifugation, then washed twice with phosphate-buffered saline (PBS). The cells were then resuspended in 100 μL 1× binding buffer, to which 5 μL of Annexin V-FITC and 5 μL of PI were added and incubated at room temperature for 15 min in the dark, after which 400 μL 1× binding buffer was added and mixed. Apoptotic cells were analyzed by a MoFlo XDP flow cytometry sorting system (Beckman Coulter, Mountain View, CA).

### Cell cycle analysis

Treated cells (1 × 10^5^ cells) were harvested and fixed with 75% ethanol at −20°C overnight, then washed twice with PBS. The cells were then resuspended in 250 μL of RNase A buffer (100 ng/mL) and incubated at room temperature for 30 min. After this incubation 2×PI (100 ng/mL) was added into the mixture and incubated for 15 min in the dark, followed by filtration with a 200 mesh filter membrane. Cell cycle was determined based on analysis on the MoFlo XDP flow cytometry sorting system (Becton Dickinson).

### Western blot analysis

Total protein was extracted by RIPA lysis buffer (CWBIO, Beijing, China) and quantified using a modified Bradford method. Equal amounts of protein samples were subjected to sodium dodecyl sulfate-polyacrylamide gel electrophoresis and transferred to a polyvinylidene fluoride membrane (Millipore Corporation, Billerica, USA). The membrane was then soaked in tris-buffered saline Tween-20 buffer (TBST) (20 mM Tris-HCl, pH 8.0, 15 0 mM NaCl, 0.05% Tween-20) with 5% bovine serum albumin (BSA), for 1 h at room temperature with gentle shaking and subsequently incubated with specific antibody against AURKA (1:1000, Cell Signaling Technology, Danvers, USA) or GAPDH (1:1000, ABclonal Technology, Wuhan, China) antibody for 2 h at room temperature or overnight at 4°C. Afterwards, the membrane was washed and incubated with horseradish peroxidase (HRP)-conjugated secondary antibody (1:10000, Signalway Antibody, Nanjing, China) for 1 h. Finally, protein bands were detected by a chemiluminescent HRP substrate (Millipore Corporation), quantitated by densitometric analysis using the Image Lab analysis software (Bio-Rad), and expressed as percentage of control after normalization to GAPDH.

### Dual luciferase reporter assay

The wild type human AURKA 3′-UTR firefly luciferase construct (pGL3-AURKA-3′-UTR-wt) was generated by inserting a 719 bp fragment of human AURKA 3′-UTR into the *Eco*R V/*Eco*R I sites of the pGL3 miReport vector. The pGL3-AURKA-3′-UTR-mut construct was generated by mutating the putative miR-32-binding site on pGL3-AURKA-3′-UTR-wt. As a control for transfection efficiency, plasmid expressing the renilla luciferase gene (pRL) was cotransfected into each transfection experiment. Cells were harvested 48 h after transfection and luciferase activity was assayed by an Orion II Microplate Illuminometer (Titertek-Berthold, South San Francisco, USA). Relative activities were expressed as the fold-change in luciferase activity after normalization to renilla luciferase activity. All constructs were sequenced to verify integrity. All the primers used in this paper are listed in Supplementary Table 2.

### Statistical analysis

Results were represented as the mean ± SEM and difference between two experimental groups was evaluated using student's *t*-test with statistical significance defined as *P* < 0.05.

## SUPPLEMENTARY MATERIALS AND METHODS


