# Supplementary material for: Tanshinones suppress AURKA through up-regulation of miR-32 expression in non-small cell lung cancer
Source: Oncotarget. 2015 May 14;6(24):20111–20. doi: 10.18632/oncotarget.3933 (PMC4652991; doi:10.18632/oncotarget.3933)
Supplement: Supplementary file 1 [file oncotarget-06-20111-s001.pdf]

SUPPLEMENTARY MATERIALS AND METHODS

Migration ability assay

The H1299 cells were treated with a series concentrations of tanshinones (T1 4 μM, T2A 4 μM, CT 5 μM, DMSO 5 μM (control)) for 48 h in 6-well micro-plates in advance. Medium contained with tanshinones (T1 4 μM, T2A 4 μM, CT 5μM, DMSO 5 μM (control); 600 μl/compartment) was added to the lower compartments of Transwell insert (24-well, 8 μm pore, PC; Corning Incorporated) and incubated for 1 h. Above treated H1299 cells were then resuspended in FBS-free medium at a concentration of 5×10^5 cells/ml and added onto the upper compartments of Transwell inserts and incubated for 24 h. After incubation, the medium was removed, and cells inside the upper compartments were cleaned by cotton buds,washing the inserts with PBS for twice. Then, the inserts were marinated in 4% paraformaldehyde solution for 15 min and following marinated in toluylene red staining solution for 15 min. Finally, washed the inserts with PBS and photoed every well with 5 stochastic views under inverted microscope (400×). The cells acrossing PC film were counted and averaged.

SUPPLEMENTARY FIGURES AND TABLES

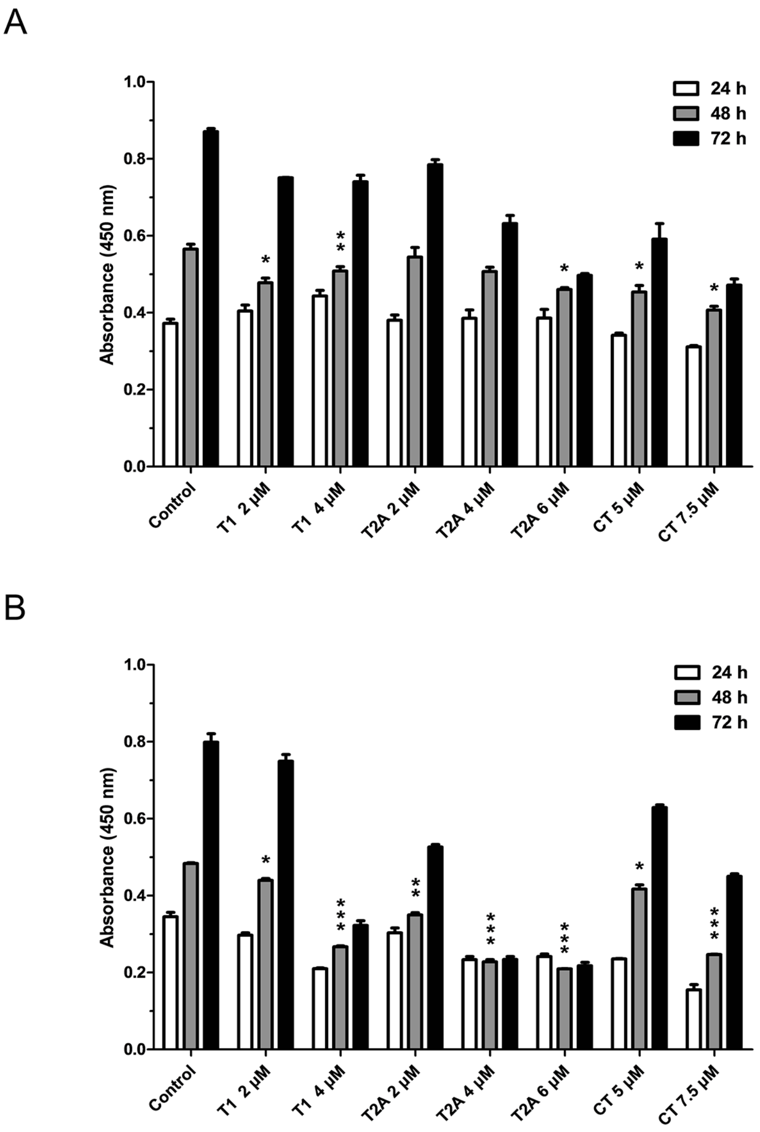

Supplementary Figure S1: Tanshinones suppress NSCLC. In **A-B,** cells were respectively treated with T1 4 μM/6 μM, T2A 2 μM/4 μM/6 μM, CT 5 μM/7.5 μM or DMSO 7.5 μM (Control) for 48 h. **A**. Cell vitality of A549 cells treated with tanshinone or DMSO was determined by CCK-8 cytotoxicity test. **B**. Cell vitality of SPCA-1 cells treated with tanshinone or DMSO was determined by CCK- 8 cytotoxicity test. Results are represented as the mean±SEM of OD_{450nm}. *$P < 0.05$, **$P < 0.001$, ***$P < 0.001$, vs. control ($n = 3$). Representative of triplicate experiments was shown.

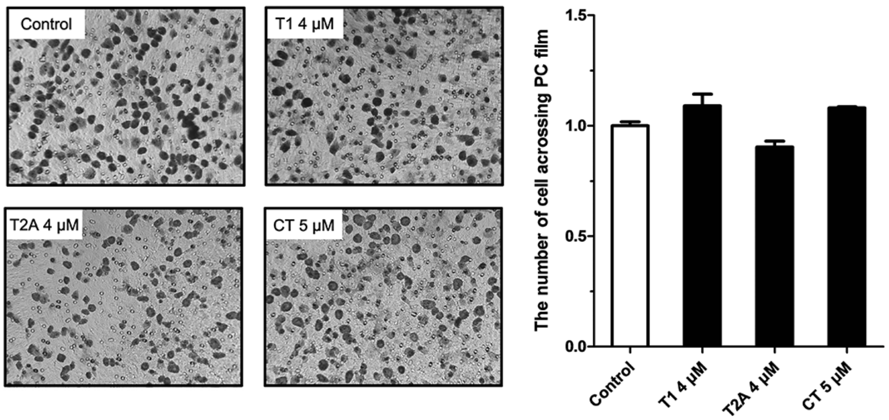

Supplementary Figure S2: Tanshinone has no effect on migration ability of H1299 cells. Migration ability of H1299 cells were measured by Transwell technique. Cells were respectively treated with T1 4 μM, T2A 4 μM, CT 5 μM or DMSO 5 μM (Control) for 48 h. Representative of triplicate experiments was shown.

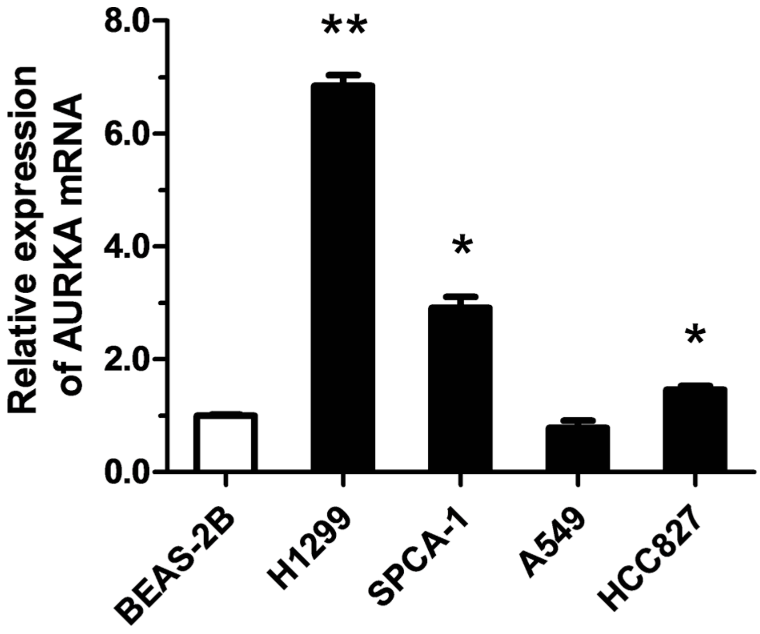

Supplementary Figure S3: The endogenous expression level of AURKA in common NSCLC cell lines. The expression levels of AURKA in lung cancer cell lines or pulmonary epithelial cell line (control) were measured by qRT-PCR. $*P < 0.05$, $**P < 0.001$, vs. control ($n = 3$). Representative of triplicate experiments was shown.

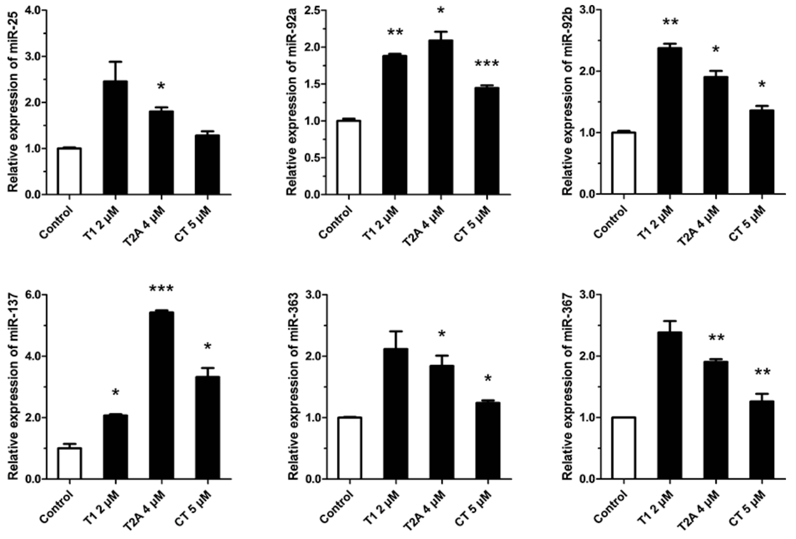

Supplementary Figure S4: The miRNAs up-regulated by Tanshinones in H1299 cells. Expression levels of miRNAs in H1299 cells respectively treated with T1 4 µM, T2A 4 µM, CT 5 µM or DMSO 5 µM (Control) for 48 h were measured by qRT-PCR. *$P < 0.05$, **$P < 0.001$, ***$P < 0.001$, vs. control ($n = 3$). Representative of triplicate experiments was shown.

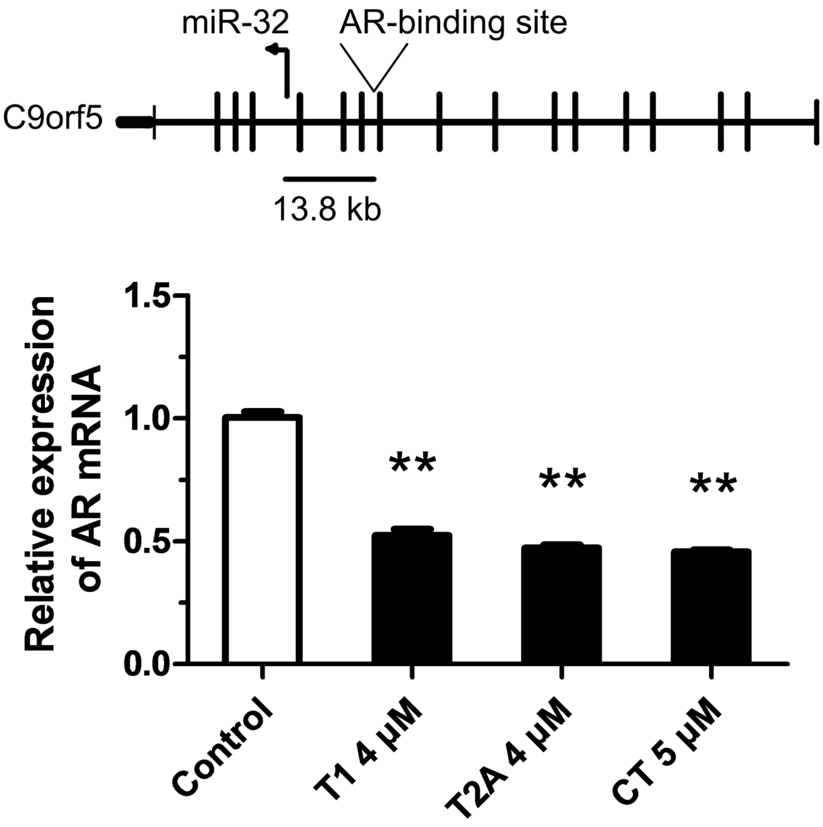

Supplementary Figure S5: Tanshinones down-regulate the expression of AR. Expression level of AR in H1299 cells respectively treated with T1 4 µM, T2A 4 µM, CT 5 µM or DMSO 5 µM (Control) for 48 h was measured by qRT-PCR. **$P < 0.001$, vs. control ($n = 3$). Representative of triplicate experiments was shown.

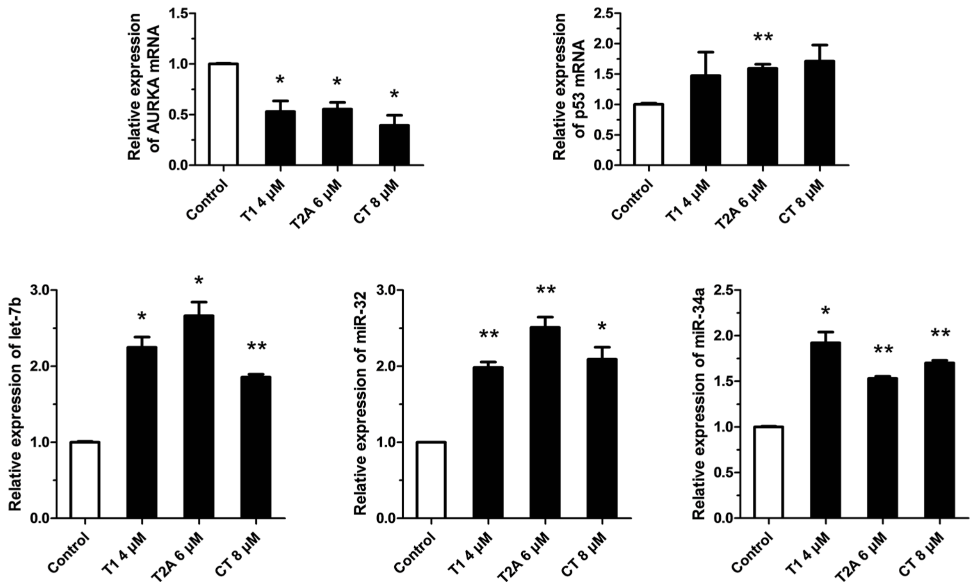

Supplementary Figure S6: The miRNAs up-regulated by Tanshinones in SPCA-1 cells. Expression levels of miRNAs in SPCA-1 cells respectively treated with T1 4 μM, T2A 6 μM, CT 8 μM or DMSO 8 μM (Control) for 48 h were measured by qRT-PCR. *$P < 0.05$, **$P < 0.001$, vs. control ($n = 3$). Representative of triplicate experiments was shown.

Supplementary Table 1: Selection of predicted miRNAs targeting AURKA

miRNA	Seed Match	Sequence	Position of AURKA 3′UTR	Oncogene/Anti-oncogene
hsa-let-7a	7	GAGGUAG	632–638	Anti-oncogene
hsa-let-7b	7	GAGGUAG	632–638	Anti-oncogene
hsa-let-7c	7	GAGGUAG	632–638	Anti-oncogene
hsa-let-7d	7	GAGGUAG	632–638	Anti-oncogene
hsa-let-7e	7	GAGGUAG	632–638	Anti-oncogene
hsa-let-7f	7	GAGGUAG	632–638	Anti-oncogene
hsa-let-7g	7	GAGGUAG	632–638	Anti-oncogene
hsa-let-7i	7	GAGGUAG	632–638	Anti-oncogene
hsa-miR-25	7	AUUGCAC	373–379	Uncertain
hsa-miR-32	8	UAUUGCAC	372–379	Anti-oncogene
hsa-miR-92a	7	AUUGCAC	373–379	Oncogene
hsa-miR-92b	7	AUUGCAC	373–379	Oncogene
hsa-miR-98	7	GAGGUAG	632–638	Anti-oncogene
hsa-miR-124	7	AAGGCAC	142–148	Anti-oncogene
hsa-miR-137	6	UAUUGC	375–380	Anti-oncogene
hsa-miR-140-5p	7	AGUGGUU	585–591	Anti-oncogene
hsa-miR-149	7	CUGGCUC	27–33	Anti-oncogene
hsa-miR-154	7	AGGUUAU	378–384	Anti-oncogene
hsa-miR-186	7	AAAGAAU	457–453	Anti-oncogene
hsa-miR-300	7	AUACAAG	495–501	Anti-oncogene
hsa-miR-363	7	AUUGCAC	373–379	Uncertain
hsa-miR-367	7	AUUGCAC	373–379	Uncertain
hsa-miR-370	7	CCUGCUG	134–140	Anti-oncogene
hsa-miR-506	7	AAGGCAC	142–148	Anti-oncogene
hsa-miR-876-5p	8	GGAUUUCU	17–24	Uncertain

Supplementary Table 2: Sequences of related primers

Primer	Sequence
U6 (F)	5′-CTCGCTTCGGCAGCACA-3′
U6 (R)	5′-AACGCTTCACGAATTTGCGT-3′
let-7b	5′-TGAGGTAGTAGGTTGTGTGGTT-3′
let-7c	5′-TGAGGTAGTAGGTTGTATGGTT-3′
miR-25	5′-CAUUGCACUUGUCUCGGUCUGA-3′
miR-32	5′-UAUUGCACAUUACUAAGUUGCA-3′
miR-34a	5′-UGGCAGUGUCUUAGCUGGUUGU-3′
miR-92a	5′-UGGCAGUGUCUUAGCUGGUUGU-3′
miR-92b	5′-UGGCAGUGUCUUAGCUGGUUGU-3′
miR-137	5′-UUAUUGCUUAAGAAUACGCGUAG-3′
miR-363	5′-AAUUGCACGGUAUCCAUCUGUA-3′
miR-367	5′-AAUUGCACUUUAGCAAUGGUGA-3′
18S (F)	5′-AAUUGCACUUUAGCAAUGGUGA-3′
18S (R)	5′-AAUUGCACUUUAGCAAUGGUGA-3′
AURKA (F)	5′-CATCTTCCAGGAGGACCACT-3′
AURKA (R)	5′-CAAAGAACTCCAAGGCTCCA-3′
AURKA 3′UTR (F)	5′-GATATCGGGAGAAATCCTTGAGCCA-3′
AURKA 3′UTR (R)	5′-AAGAATTCCACACATGCTATGACTCCAATGT-3′
AURKA 3′UTR Mut (F)	5′-AAGGAGCAGAGGATTAACCTTCCTAGTACCTGAGTGAGT-3′
AURKA 3′UTR Mut (R)	5′-AAGGTTAATCCTCTGCTCCTTAACTGATCGGGGTCA-3′
AR (F)	5′-CTATTGCGAGAGAGCTGCAT-3′
AR (R)	5′-CAATAGAGGAAATTCCCCAA-3′
